# Circulating EZH2-positive T cells are decreased in multiple sclerosis patients

**DOI:** 10.1186/s12974-018-1336-9

**Published:** 2018-10-26

**Authors:** Sunny Malhotra, Luisa M. Villar, Carme Costa, Luciana Midaglia, Marta Cubedo, Silvia Medina, Nicolás Fissolo, Jordi Río, Joaquín Castilló, José C. Álvarez-Cermeño, Alex Sánchez, Xavier Montalban, Manuel Comabella

**Affiliations:** 1 Servei de Neurologia-Neuroimmunologia, Centre d’Esclerosi Múltiple de Catalunya (Cemcat), Institut de Recerca Vall d’Hebron (VHIR), Hospital Universitari Vall d’Hebron, Universitat Autònoma de Barcelona, Barcelona, Spain; 2 Departments of Neurology and Immunology, Hospital Universitario Ramón y Cajal, Instituto Ramón y Cajal de Investigacion Sanitaria, Madrid, Spain; 3 Departament d’Estadística, Facultat de Biologia, Universitat de Barcelona, Barcelona, Spain; 4 Unitat d’Estadística i Bioinformàtica, Institut de Recerca, HUVH, Barcelona, Spain; 5 Genetics, Microbiology and Statistics Department, Universitat de Barcelona, Barcelona, Spain

**Keywords:** Multiple sclerosis, EZH2, Treatment, Migration, Adhesion molecules

## Abstract

**Background:**

Recent studies in experimental autoimmune encephalomyelitis, an animal model of multiple sclerosis (MS), suggest an involvement of the histone methyltransferase enhancer of zeste 2 polycomb repressive complex 2 subunit (EZH2) in important processes such as cell adhesion and migration.

**Methods:**

Here, we aimed to expand these initial observations by investigating the role of EZH2 in MS. mRNA expression levels for EZH2 were measured by real-time PCR in peripheral blood mononuclear cells (PBMC) from 121 MS patients (62 untreated and 59 receiving treatment) and 24 healthy controls.

**Results:**

EZH2 expression levels were decreased in PBMC from untreated patients compared to that from controls, and treatment significantly upregulated EZH2 expression. Expression of miR-124 was increased in MS patients compared to controls. Blood immunophenotyping revealed EZH2 expression mostly restricted to CD4+ and CD8+ T cells, and circulating EZH2+ CD4+ and CD8+ T cells were decreased in untreated MS patients compared to controls. CD8+ T cells expressing EZH2 exhibited a predominant central memory phenotype, whereas EZH2+ CD4+ T cells were of effector memory nature, and both T cell subsets produced TNF-α. EZH2+ T cells were enriched in the cerebrospinal fluid compartment compared to blood and were found in chronic active lesions from MS patients. EZH2 inhibition and microarray analysis in PBMC was associated with significant downregulation of key T cell adhesion molecules.

**Conclusion:**

These findings suggest a role of EZH2 in the migration of T cells in MS patients. The observation of TNF-α expression by CD4+ and CD8+ T cells expressing EZH2 warrants additional studies to explore more in depth the pathogenic potential of EZH2+-positive cells in MS.

**Electronic supplementary material:**

The online version of this article (10.1186/s12974-018-1336-9) contains supplementary material, which is available to authorized users.

## Background

Enhancer of zeste 2 polycomb repressive complex 2 subunit (EZH2) is a histone methyltransferase that serves as the catalytic subunit of the polycomb repressive complex 2, a protein complex that regulates gene expression by methylating nucleosomal histone H3 at lysine 27 (H3K27) on the promoter of its target genes [1]. The identification of a cytosolic methyltransferase EZH2-containing complex suggested that, in addition to its role methylating histones, EZH2 could also be involved in the regulation of extra-nuclear signaling pathways, in particular actin polymerization-dependent processes [2]. Recent investigation on its cytoplasmic role has revealed that EZH2 interacts with cytosolic proteins such as talin and the guanine nucleotide–exchange factor vav1 that link integrin molecules to the actin cytoskeleton, suggesting the potential implication of EZH2 in cell adhesion and migration processes [3]. Interestingly, mice lacking the EZH2 gene exhibited attenuated experimental autoimmune encephalomyelitis (EAE) disease progression due to the inability of EZH2-deficient cells, particularly neutrophils and dendritic cells, to reach the site of inflammation [3]. Taking into consideration the findings of EZH2 in EAE mice and its implication in important processes for the pathogenesis of multiple sclerosis such as cell adhesion and migration, we believe that EZH2 may also be playing a role in multiple sclerosis and contribute to the inflammatory component observed in the central nervous system (CNS) of patients. Hence, the purpose of the present study was to explore the role of EZH2 in the disease by measuring the gene expression levels of EZH2 and associated molecules in peripheral blood cells from untreated and treated multiple sclerosis patients and by characterizing the immune cell populations responsible for EZH2 expression.

## Methods

### Patients

#### Initial cohort

Messenger RNA (mRNA) expression levels of EZH2, talin 1 (TLN1), and VAV1 were determined in peripheral blood mononuclear cells (PBMC) from a first cohort of 24 healthy controls (HC) and 62 treatment-naïve multiple sclerosis patients. The case group included 25 patients with relapsing-remitting multiple sclerosis (RRMS), 20 patients with secondary progressive multiple sclerosis (SPMS), and 17 patients with primary progressive multiple sclerosis (PPMS). The RRMS group included 20 patients in clinical remission and 5 patients whose blood was drawn at the time of an acute relapse.

#### Validation cohort

In order to replicate EZH2 findings, mRNA expression levels for EZH2 were also measured in PBMC from an independent validation cohort comprised of 12 HC and 13 treatment-naïve multiple sclerosis patients. Considering that EZH2 expression levels in the initial cohort were similar between different clinical forms of the disease, for the validation cohort, only patients with RRMS were included.

#### Treated cohort

EZH2 and TLN1 mRNA expression levels were determined in an additional cohort of 59 RRMS patients treated for at least 1 year with interferon-beta (*n* = 17), glatiramer acetate (*n* = 15), fingolimod (*n* = 16), or natalizumab (*n* = 11). Expression levels for these genes were compared with those observed in a subgroup of 14 untreated RRMS patients included in the initial cohort.

The study was approved by the local Ethics Committee [EPA(AG)57/2013(3834)], and participants gave written informed consent. Tables 1 and 2 summarize demographic and baseline clinical characteristics of multiple sclerosis patients from the initial, validation, and treated cohorts and the HC included in the study.

**Table 1:** Demographic and baseline clinical characteristics of the MS patients and healthy controls

Baseline characteristics	HC	RRMS	SPMS	PPMS	Relapse
Initial cohort					
*N*	24	20	20	17	5
Age (years)	30.2 (7.2)	30.0 (7.8)	45.7 (8.7)	50.2 (7.3)	30.8 (8.7)
Female/male (% women)	18/6 (75.0)	10/10 (50.0)	11/9 (55.0)	11/6 (64.7)	2/3 (40.0)
Duration of disease (years)	–	4.8 (4.6)	11.5 (7.6)	12.2 (7.9)	2.2 (2.7)
EDSS^a^	–	1.7 (1.0–4.2)	4.0 (3.5–5.1)	6.0 (4.0–6.0)	3.0 (2.5–5.3)
Numbers of relapses^b^	–	2.2 (0.7)	0.8 (0.8)	–	2.6 (1.3)
Validation cohort					
*N*	12	13			
Age (years)	28.2 (6.0)	37.6 (9.3)			
Female/male (% women)	8/3 (72.7)	12/2 (85.7)			
Duration of disease (years)	–	3.8 (3.2)			
EDSS^a^	–	2.5 (1.0–3.5)			
Numbers of relapses^b^	–	2.5 (0.8)			

Data are expressed as mean (standard deviation) unless otherwise stated

*RRMS* relapsing-remitting multiple sclerosis, *SPMS* secondary progressive multiple sclerosis, *PPMS* primary progressive multiple sclerosis, *Relapse* RRMS patients whose blood was collected at the time of an acute exacerbation

^a^Data are expressed as mean (interquartile range)

^b^The number of relapses in the 2 years before blood collection

**Table 2:** Summary of demographic and baseline clinical characteristics of the treated MS cohort

Characteristics	UNT	IFN	GA	FG	NTZ
*N*	14	17	15	16	11
Age (years)	28.3 (6.3)	34.8 (7.5)	32.3 (7.9)	30.3 (7.8)	27.7 (14.5)
Female/male (% women)	8/6 (57.2)	9/8 (52.9)	8/7 (53.3)	11/5 (68.7)	7/4 (63.6)
Duration of disease (years)	3.3 (2.7)	5.0 (10.7)	6.7 (5.8)	3.0 (3.8)	6.1 (7.0)
EDSS^a^	1.8 (1.4–2.5)	1.6 (1.0–2.0)	2.2 (1.5–3.0)	1.6 (1.0–2.0)	2.5 (1.6–3.5)
Numbers of relapses^b^	2.0 (0.8)	1.5 (0.8)	2.3 (1.5)	2.2 (0.7)	1.9 (0.6)

Data are expressed as mean (standard deviation) unless otherwise stated

*UNT* untreated relapsing-remitting MS patients, *IFN* interferon-beta, *GA* glatiramer acetate, *FG* fingolimod, *NTZ* natalizumab

^a^Data are expressed as mean (interquartile range) and refers to EDSS at the time of treatment onset

^b^The number of relapses in the 2 years before treatment onset

### Sample collection and determination of mRNA expression levels of EZH2, TLN1, and VAV1 by real-time PCR

PBMC from multiple sclerosis patients and HC were isolated by Ficoll-Isopaque density gradient centrifugation (Gibco BRL, Life Technologies LTD, UK) and stored in liquid nitrogen until used. Total RNA was extracted from PBMC using an RNeasy kit (Quiagen, Santa Clarita, USA) and cDNA synthesized using the High-Capacity cDNA Archive kit (Applied Biosystems, Foster City, CA, USA). mRNA expression levels for EZH2, TLN1, and VAV1 were determined with TaqMan® probes specific for the gene (Applied Biosystems). The housekeeping gene glyceraldehyde-3-phosphate dehydrogenase (GAPDH) was used as an endogenous control (Applied Biosystems). Assays were run on the ABI PRISM® 7900HT system (Applied Biosystems), and data were analyzed with the 2^−ΔΔCT^ method [4].

### Determination of microRNA expression levels by real-time PCR

Expression levels for miR-124 and miR-155 were determined according to sample availability in PBMC from a subgroup of 18 HC and 21 untreated multiple sclerosis patients (15 RRMS and 6 SPMS patients) who were also included in the initial cohort. Additional file 1: Table S1 summarizes demographic and main clinical characteristics of individuals included for this part of the study. PBMC were collected and processed in the same conditions as described in the previous section. Expression levels for miR-124 and miR-155 were measured with TaqMan® probes specific for the microRNAs (Applied Biosystems) using RNU 6b as endogenous control. Analysis was performed as described above with the 2^−ΔΔCT^ method [4].

### EZH2 immunophenotyping

EZH2 protein expression was determined by flow cytometry according to sample availability in PBMC from 13 HC [9 females (69.2%); mean age (standard deviation), 35.8 years (10.9)] and 10 RRMS patients [5 females (50%); mean age, 32.1 years (13.5); mean disease duration, 4.5 (3.5)] at baseline and after 1 year of natalizumab treatment. Only one MS patient and one HC were also included in the initial cohort whereas the remaining individuals corresponded to new multiple sclerosis patients and HC. EZH2 expression was also determined in cerebrospinal fluid (CSF) cells from 3 untreated RRMS patients [2 females (66.7%); mean age, 34.3 years (11.9); mean disease duration, 0.4 (0.5)]. CSF samples were collected by lumbar puncture for clinical purposes and centrifuged at 1200*g* for 15 min. Supernatants were stored at − 80 °C until processed for clinical tests and CSF cells resuspended in PBS and labeled as described below.

### Monoclonal antibodies

The following monoclonal antibodies were used in the study: EZH2-Alexa Fluor 488, CD197-PE (CCR7-PE), CD3-PE, granulocyte/macrophage colony-stimulating factor (GM-CSF)-PE, CD16-PE-Cy5, tumor necrosis factor (TNF)-α-PercP-Cy5.5, CD19-PE-Cy7, CD45RO-APC, CD56-APC, CD8-APC-H7, CD14-APC-H7, CD3-BV421, CD45-V450, CD45-V500 (all from BD Biosciences, San Diego, CA), and IL-17-APC (R&D Systems, Minneapolis, MN).

### Characterization of EZH2 expression by CSF cells

CSF cells were stained for 30 min at 4 °C in the dark with the appropriate amounts of monoclonal antibodies recognizing the surface antigens. Subsequently, cells were washed with PBS, fixed and permeabilized for 20 min at 4 °C in the dark with Cytofix/Cytoperm Kit (BD Biosciences), washed twice with Perm/Wash solution (BD Biosciences) and stained intracellularly for 30 min at 4 °C in the dark with a monoclonal antibody recognizing EZH2, and washed and analyzed in a FACSCanto II flow cytometer (BD Biosciences).

### Intracellular cytokine staining

Aliquots of 10^6^ PBMC were resuspended in 1 ml of complete medium with 50 ng/ml phorbol 12-myristate 13-acetate (PMA) (Sigma-Aldrich, St. Louis, MO) and 750 ng/ml ionomycin (Sigma-Aldrich), in the presence of 2 μg/ml brefeldin A (GolgiPlug, BD Biosciences) and 2.1 μM monensin (Golgi Stop, BD Biosciences) in polypropylene tubes, and incubated for 4 h at 37 °C in 5% CO_2_. Cells were washed in PBS and surface stained as indicated above. Afterward, cells were fixed and permeabilized for 20 min at 4 °C in the dark with Cytofix/Cytoperm Kit (BD Biosciences), washed twice with Perm/Wash solution (BD Biosciences), and stained with monoclonal antibodies recognizing GM-CSF, TNF-α, and IL-17.

### Flow cytometry analysis

Cells were always analyzed within 1 h of staining. Mean autofluorescence values were set using appropriate negative isotype controls. Data analysis was performed using FACSDiva Software V.8.0 (BD Biosciences). A gate including lymphocytes and monocytes and excluding debris and apoptotic cells was established; a minimum amount of 30,000 events for PBMC samples and 500 events for CSF cells were analyzed.

### EZH2 expression in EAE mice

Anesthetized C57BL/6 mice were immunized by subcutaneous injections of PBS containing 50 μg of MOG_35–55_ (Proteomics Section, Universitat Pompeu Fabra, Barcelona, Spain) or PBS, emulsified in complete Freund’s adjuvant (Sigma Chemical, St. Louis, MO, USA), and supplemented with 2 mg/ml *Mycobacterium tuberculosis* H37RA (Difco Laboratories, Detroit, MI, USA). The animals received an additional intravenous injection of 150 ng pertussis toxin in 100 μl PBS on the day of immunization and again 48 h later. Four animals per group (EAE or controls—PBS) were sacrificed at 8, 16, 22, 29, 36, and 50 days post-immunization, and spinal cord tissue was subsequently obtained. mRNA expression levels of EZH2 and CD3e were determined by real-time PCR as previously described. Changes in gene expression were always compared with animals treated with PBS at the respective days.

### EZH2 expression in human brain tissue

#### Samples

Paraffin-embedded brain samples from RRMS patients and non-neurological controls were provided by the UK Multiple Sclerosis Tissue Bank. Tissue sections were stained with hematoxylin and eosin (HE) and Klüver-Barrera (KB) for inflammation and demyelination assessment. Ten samples from multiple sclerosis patients with chronic active lesions and four control samples were selected for the study (demographic and clinical information was not available for these patients).

#### Immunohistochemistries

Immunostainings were developed with the automated Benchmark XT platform from Ventana Medical System. Briefly, 4-μm-thick, paraffin-embedded serial sections were deparaffinized with EZ prepTM (Ventana Medical System). Antigen retrieval was performed with Cell Conditioning 1 pH = 8 (Ventana Medical System) for 30 min. Endogenous peroxidase activity was blocked with hydrogen peroxide 3%. Samples were incubated with rabbit anti-EZH2 (clone EPR9307(2), Abcam) for 36 min and visualized with ultraView Universal DAB (Ventana Medical Systems). Subsequently, samples were kept at 95 °C for 8 min and incubated with rabbit anti-CD4 (clone SP35, Ventana Medical System) or rabbit anti-CD8 (clone SP57, Ventana Medical System) for 40 min and visualized with ultraView Universal Alkaline Phosphatase Red Detection (Ventana Medical Systems). All samples were counterstained with hematoxylin.

#### Immunostaining assessment

A range between 5 and 20 pictures were taken for each multiple sclerosis sample. Total CD4+ and CD8+ T cells and double-positive EZH2 and CD4 or CD8 cells were counted. The percentages of double-positive cells were calculated with respect to the total of CD4+ or CD8+ T cells.

### EZH2 blocking and gene expression microarrays

PBMC from 7 untreated RRMS patients [5 females (71.4%); mean age, 39.0 years (8.0); mean disease duration, 7.0 years (5.1)] were plated into 24-well plates for 24 h in the presence or absence of an EZH2 inhibitor (histone deacetylase inhibitor suberoylanilide hydroxamic acid—SAHA) at 1 μg/μl concentration. After 24 h, cells were harvested and total RNA isolated using the RNeasy kit (Quiagen) and hybridized to Affymetrix Human Transcriptome Arrays (HTA 2.0) (Affymetrix, Santa Clara, CA, USA) according to the manufacturer’s protocol (GeneChip WT Pico Reagent Kit (Affymetrix)).

### Statistical analysis

Statistical analysis was performed by using the SPSS 17.0 package (SPSS Inc., Chicago, IL) for MS Windows. Comparisons of mRNA expression levels for EZH2, TLN1, and VAV1; expression levels for miR-124 and miR-155; and the percentage of EZH2-positive cells between the different study groups were performed by parametric and non-parametric tests depending on the applicability conditions. Real-time PCR data were expressed as fold change in gene expression in controls relative to the whole group of multiple sclerosis patients and patients stratified according to the different clinical forms, in RRMS patients in clinical remission relative to patients in relapse, and in treated RRMS relative to untreated patients. For microarray analysis, images were processed with AGCC, Affymetrix GeneChip Command Console, to generate .CEL files. Raw expression values obtained directly from .CEL files were pre-processed using the RMA method [5]. These normalized values were the basis for all the subsequent analyses. Previous to any analysis data were submitted to non-specific filtering to remove low-signal genes (those genes whose mean signal in each group did not exceed a minimum threshold) and low-variability genes (those genes whose standard deviation between all samples did not exceed a minimum threshold). The selection of differentially expressed genes between the untreated and the EZH2 blocking conditions was based on a linear model analysis with empirical Bayes moderation of the variance estimates following the methodology developed by Smyth [6]. In order to deal with the multiple testing issues derived from the fact that many tests (one per gene) were performed simultaneously, *p* values were adjusted to obtain strong control over the false discovery rate using the Benjamini and Hochberg method [7].

## Results

### EZH2 expression is decreased in multiple sclerosis patients

**Fig. 1:**
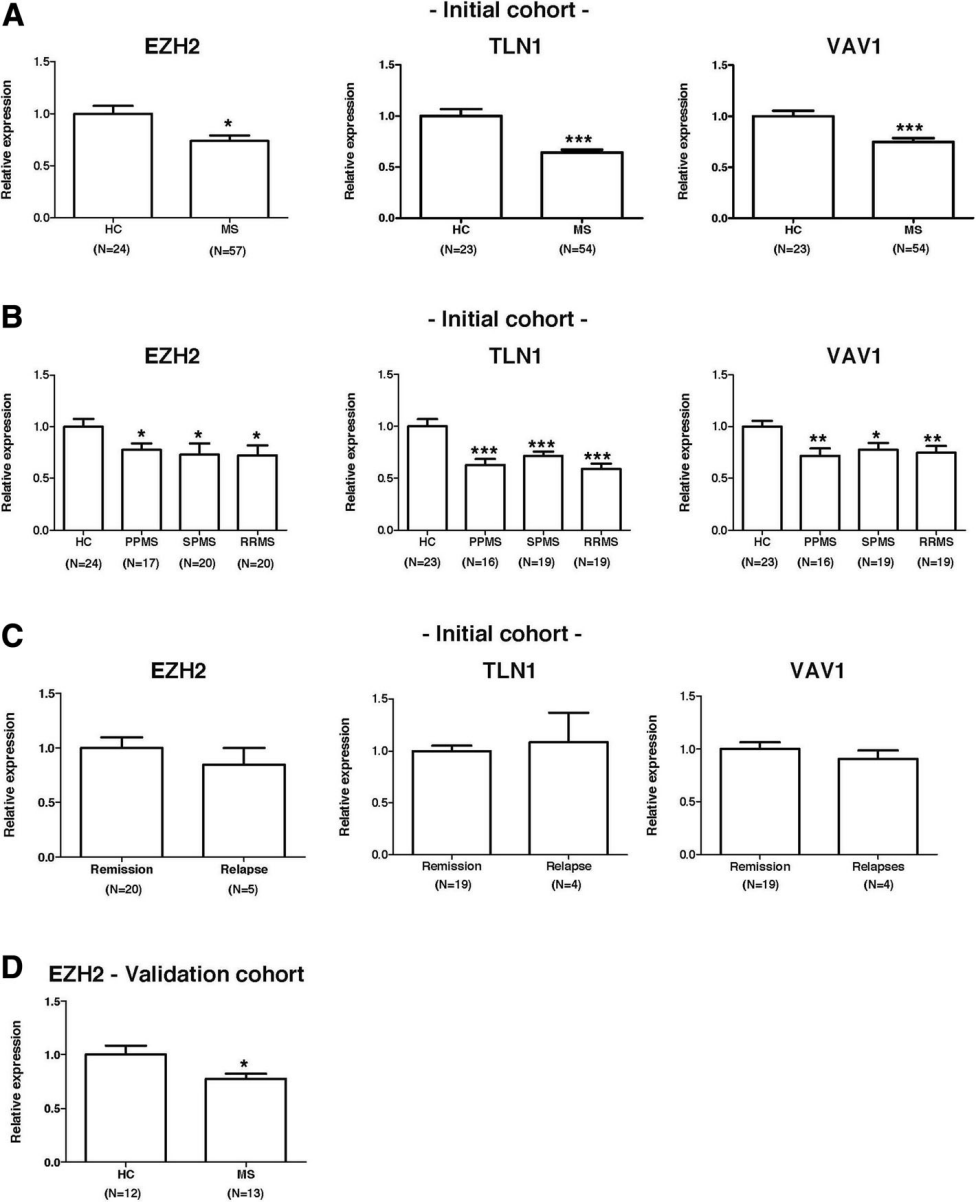
Expression levels of EZH2 and EZH2-associated molecules in multiple sclerosis patients and controls. mRNA expression levels for EZH2, TLN1, and VAV1 were determined in PBMC from untreated multiple sclerosis patients and healthy controls by real-time PCR relative quantification, as described in the “Methods” section. Graphs showing expression levels for EZH2, TLN1, and VAV1 **a**–**c** in the initial discovery cohort and **d** in an independent cohort of patients and controls. Results are expressed as fold change (standard error of the mean) in gene expression in multiple sclerosis patients relative to controls and in patients in relapse relative to patients in remission. Statistics: unpaired Student’s *t* test. *p values < 0.05; **p values < 0.01; ***p values < 0.001. HC healthy controls, MS whole group of multiple sclerosis patients, RRMS relapsing-remitting multiple sclerosis, SPMS secondary progressive multiple sclerosis, PPMS primary progressive multiple sclerosis, Remission RRMS patients in clinical remission, Relapse RRMS patients whose blood was collected at the time of an acute exacerbation, EZH2 enhancer of zeste 2 polycomb repressive complex 2 subunit, TLN1 talin 1, VAV1 vav guanine nucleotide exchange factor 1

In order to investigate the role of EZH2 in multiple sclerosis, we first measured the mRNA expression levels of EZH2 and EZH2-associated genes in PBMC from an initial cohort of 62 untreated multiple sclerosis patients and 24 HC. As shown in Fig. 1a, expression levels for EZH2, TLN1, and VAV1 were significantly decreased in PBMC from the whole multiple sclerosis group compared to controls. Further stratification of the multiple sclerosis group into the different clinical forms revealed significantly decreased gene expression levels of EZH2, TLN1, and VAV1 in PBMC from RRMS, SPMS, and PPMS patients compared to HC (Fig. 1b). As depicted in Fig. 1c, mRNA expression levels for EZH2, TLN1, and VAV1 were not changed in RRMS patients at the time of acute exacerbations, and expression levels for these genes were similar between RRMS patients in clinical remission and RRMS patients during relapse.

EZH2 findings were validated in an independent cohort of 13 untreated multiple sclerosis patients and 12 HC, and mRNA expression levels for EZH2 were again found to be significantly decreased in PBMC from the multiple sclerosis group compared to the HC group (*p* = 0.01; Fig. 1d).

### Expression levels of miR-124 are increased in multiple sclerosis patients

**Fig. 2:**
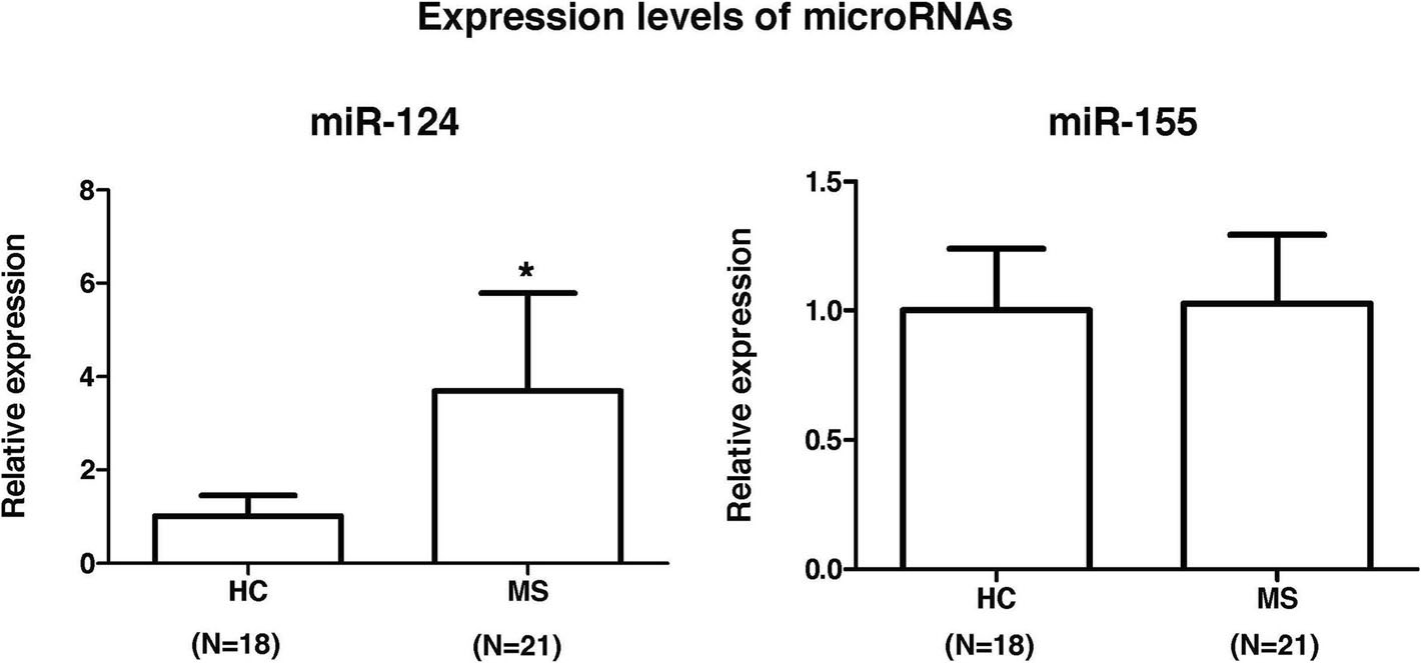
Expression levels of miR-124 and miR-155 in multiple sclerosis patients and controls. Expression levels for miR-124 and miR-155 were determined in PBMC from untreated multiple sclerosis patients and controls by real-time PCR relative quantification. Results are expressed as fold change (standard error of the mean) in gene expression in patients relative to controls. Statistics: unpaired Student’s *t* test. *p value = 0.03. HC healthy controls, MS whole group of untreated multiple sclerosis patients, which included 15 RRMS and 6 SPMS patients

We next investigated the expression levels of miR-124 and miR-155, two microRNAs that are known on the one hand to target EZH2 [8, 9] and on the other hand to be involved in multiple sclerosis [10, 11]. Following the determination of microRNA expression levels in 21 untreated multiple sclerosis patients and 18 HC, miR-124 expression was found to be significantly upregulated in PBMC from multiple sclerosis patients compared to controls (*p* = 0.03), whereas miR-155 expression levels were similar between patients and HC (Fig. 2). These results may suggest a potential and inverse relationship between EZH2 and miR-124 expression levels in multiple sclerosis patients.

### EZH2 expression is increased in treated multiple sclerosis patients

**Fig. 3:**
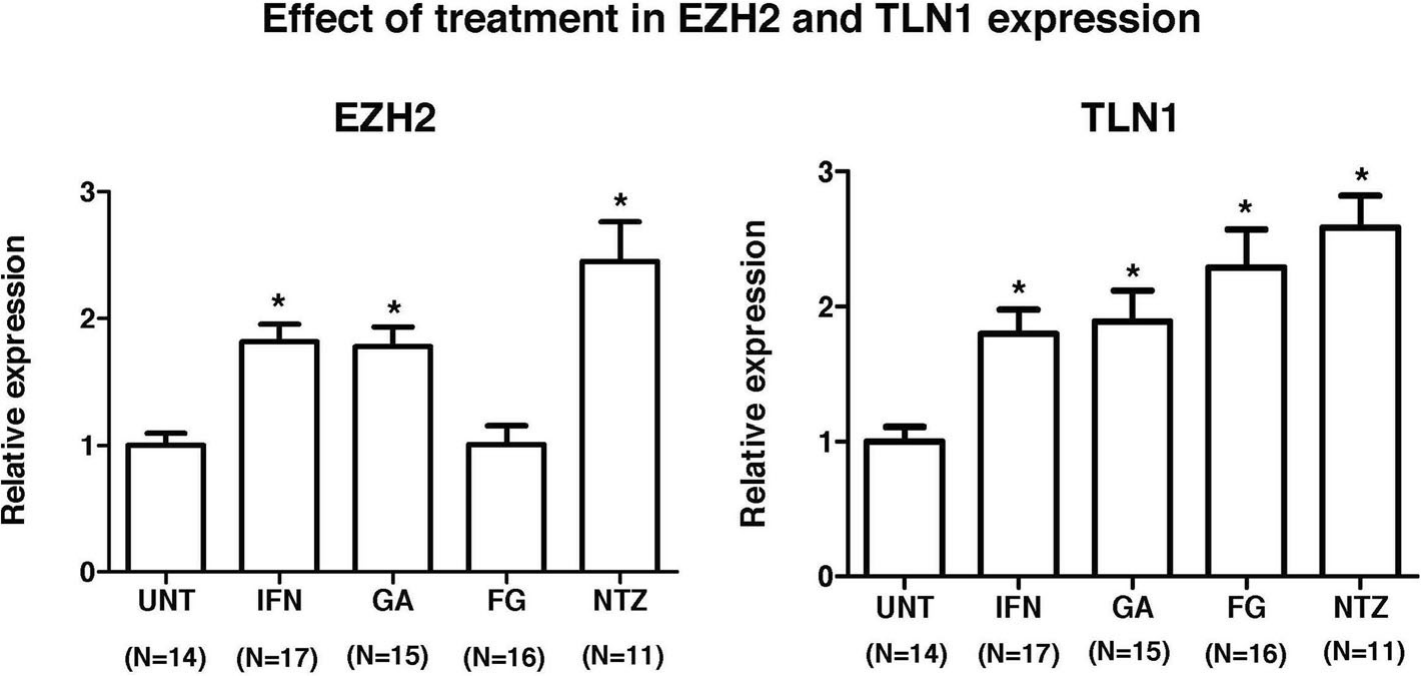
Expression levels of EZH2 and TLN1 in treated multiple sclerosis patients. mRNA expression levels for EZH2 and TLN1 were determined by real-time PCR relative quantification in PBMC from untreated multiple sclerosis patients and patients receiving disease-modifying therapies for at least 1 year. Results are expressed as fold change (standard error of the mean) in gene expression in patients relative to controls. Statistics: Mann-Whitney *U* test. *p values < 0.001. UNT untreated patients, IFN interferon-beta, GA glatiramer acetate, FG fingolimod, NTZ natalizumab, EZH2 enhancer of zeste 2 polycomb repressive complex 2 subunit, TLN1 talin 1

As a next step, we investigated whether EZH2 and TLN1 expression was modulated by commonly used multiple sclerosis therapies. For this, mRNA expression levels for EZH2 and TLN1 were determined in PBMC from 59 treated patients. Compared to untreated patients, EZH2 and TLN1 expression was significantly upregulated in PBMC by the effect of interferon-beta, Copaxone, and natalizumab treatments (Fig. 3). In contrast, whereas fingolimod significantly increased TLN1 expression, this treatment had no effect on EZH2 expression (Fig. 3). Overall, the increased EZH2 and TLN1 expression in PBMC from treated MS patients may indicate a reduced leukocyte trafficking into the CNS by the effect of treatment.

### EZH2 is expressed by circulating CD4+ and CD8+ T cells with effector memory and central memory phenotypes respectively

**Fig. 4:**
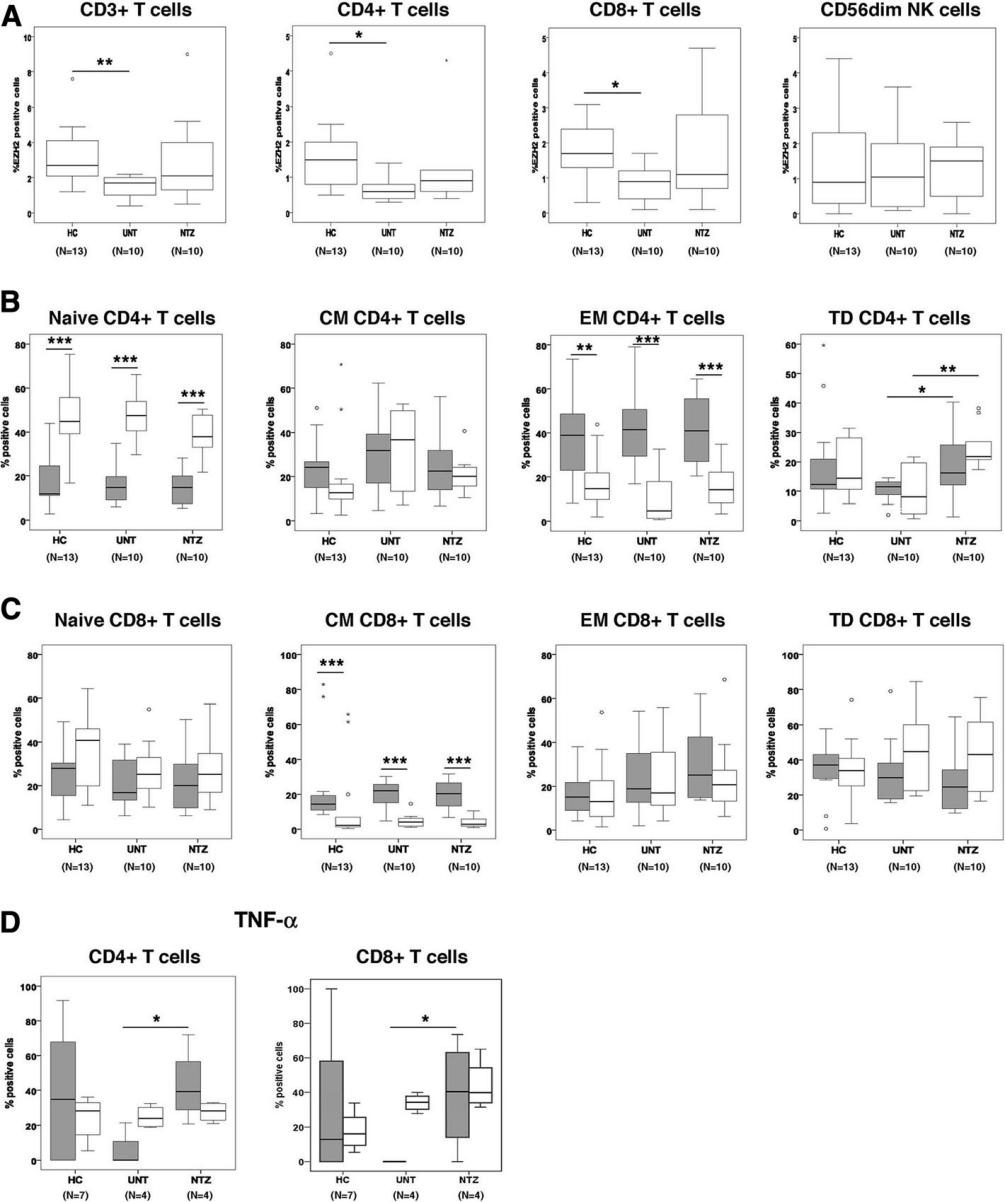
EZH2 immunophenotyping in PBMC from multiple sclerosis patients and controls. **a** Graphs showing the percentage of EZH2-positive cells in T cells and CD56dim NK cells from healthy controls (HC; *n* = 13), untreated multiple sclerosis patients (UNT; *n* = 10), and the same cohort of patients after 1 year of natalizumab treatment (NTZ; *n* = 10). **b**, **c** Graphs showing EZH2 expression in different CD4+ and CD8+ T cell subsets (HC, *n* = 13; UNT, *n* = 10; NTZ, *n* = 10). Naïve T cells were defined as CD45RO−/CCR7+. Central memory (CM) T cells were defined as CD45RO+/CCR7+. Effector memory (EM) T cells were defined as CD45RO+/CCR7−. Terminally differentiated (TD) effector T cells were defined as CD45RO−/CCR7+. Gray boxes, EZH2-positive cells; open boxes, EZH2-negative cells. **d** Graphs showing the percentage of tumor necrosis factor (TNF)-α-positive cells in CD4+ and CD8+ T cells expressing EZH2 (HC, *n* = 7; UNT, *n* = 4; NTZ, *n* = 4). Gray boxes, EZH2-positive cells; open boxes, EZH2-negative cells. Statistics: **a**, **b** unpaired Student’s *t* test; **c**, **d** Mann-Whitney *U* test. *p values < 0.05; **p values < 0.01; ***p values < 0.001. EZH2 enhancer of zeste 2 polycomb repressive complex 2 subunit

In order to characterize the PBMC populations that express EZH2, immunophenotyping for EZH2 and flow cytometry analysis was performed in T cells (CD3+, CD4+, and CD8+), B cells, monocytes, and NK cells from 10 multiple sclerosis patients and 13 HC. EZH2 expression was restricted to CD3+ (both CD4+ and CD8+) T cells and CD56dim NK cells (Fig. 4a), whereas it was absent in B cells and monocytes. Similar to the mRNA expression findings observed in the whole PBMC population, the percentage of EZH2-positive cells in CD4+ and CD8+ T cells was significantly reduced in untreated multiple sclerosis patients compared to controls (Fig. 4a). Although treatment with natalizumab, which was selected as control therapy, increased EZH2 expression by T cells, differences did not reach statistical significance (Fig. 4a). In contrast, EZH2 expression by CD56dim NK cells was similar across the different groups (Fig. 4a).

Further, EZH2 immunophenotyping in naïve and different memory T cell populations revealed that CD4+ T cells expressing EZH2 had a clear effector memory phenotype with low contribution of naïve T cells compared to CD4+ T cells negative for EZH2 expression (Fig. 4b). In contrast, CD8+ T cells expressing EZH2 exhibited a predominant central memory phenotype compared to EZH2-negative CD8+ T cells (Fig. 4c). A trend towards decreased expression of EZH2 (*p* = 0.07) was observed in terminally differentiated effector CD4+ T cells from untreated multiple sclerosis patients compared to HC, and EZH2 expression was significantly upregulated in patients by the effect of natalizumab treatment (*p* = 0.03) (Fig. 4b). However, a similar pattern was also observed in terminally differentiated effector CD4+ T cells negative for EZH2 (*p* = 0.05 and *p* = 0.003 in untreated patients versus controls and patients receiving treatment respectively) (Fig. 4b).

Finally, in order to evaluate the pathogenic potential of EZH2-positive cells, staining for proinflammatory cytokines such as TNF-α, GM-CSF, and IL-17 was also included in CD4+ and CD8+ T cells from a subgroup of untreated (*N* = 4) and treated (*N* = 4) patients and HC (*N* = 7). EZH2-positive cells expressed TNF-α though were negative for GM-CSF and IL-17 expression. Interestingly, trends towards decreased percentage of TNF-α-positive cells were observed in CD4+ and CD8+ T cells expressing EZH2 from untreated patients compared to HC (*p* = 0.08 and *p* = 0.07 respectively), whereas no similar findings were seen in their EZH2-negative counterparts (Fig. 4d). Furthermore, natalizumab treatment was associated with significant increases in the percentage of TNF-α-positive cells in CD4+ and CD8+ T cells expressing EZH2 (*p* = 0.02 and *p* = 0.04 respectively), while treatment had no effect in the percentage of TNF-α-positive cells by EZH2-negative CD4+ and CD8+ T cells (Fig. 4d).

Altogether, these data point to a different expression of EZH2 depending on the differentiation stages of the CD4+ and CD8+ T cells and suggest a common pathogenic potential of EZH2-positive cells in MS via TNF-α production.

### EZH2-positive T cells migrate to the CNS during EAE and multiple sclerosis

**Fig. 5:**
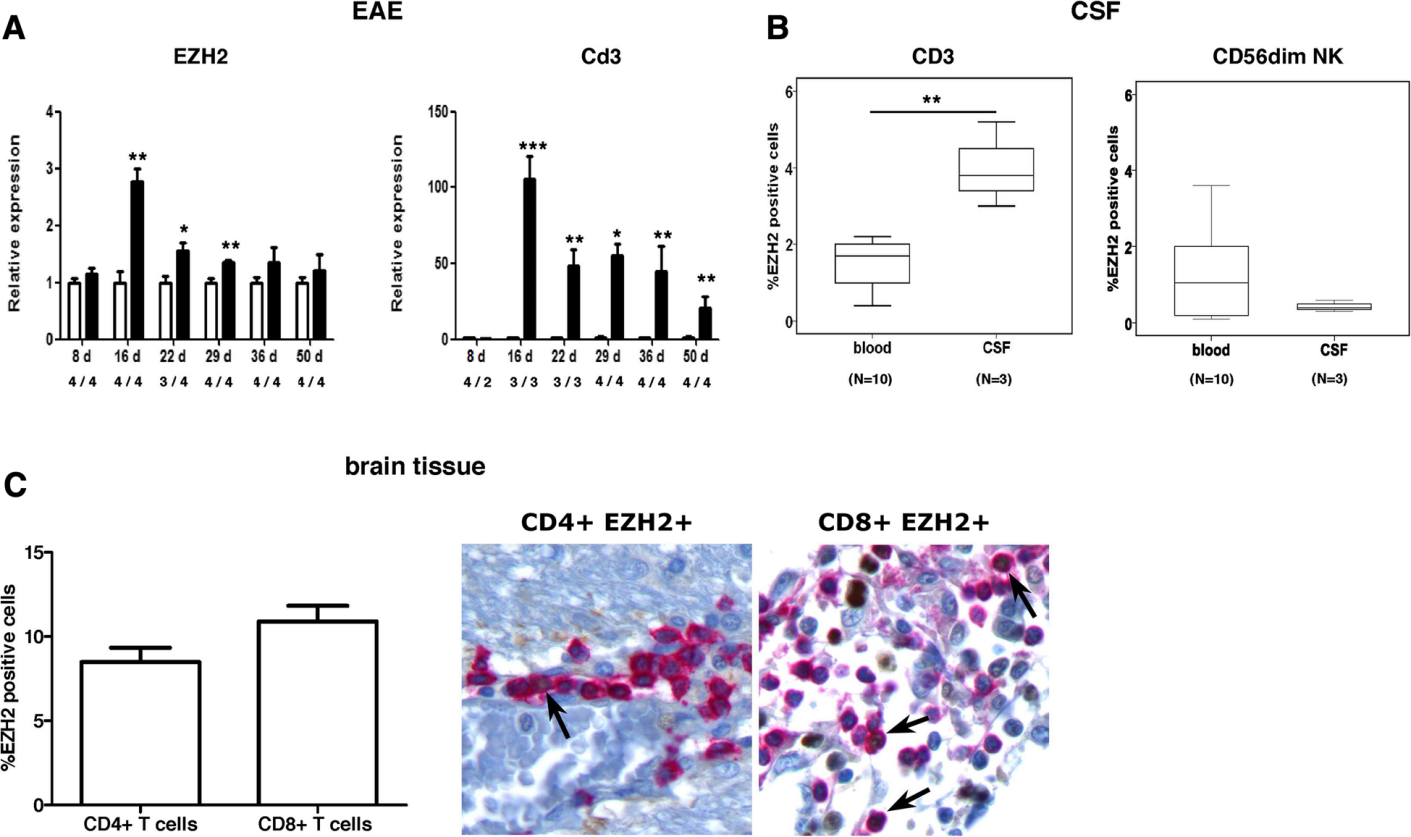
EZH2 expression in the CNS of EAE and multiple sclerosis patients. **a** Graphs showing expression levels for EZH2 and Cd3 in spinal cord from EAE mice (black bars) and control mice (open bars). The *x*-axis indicates days post-immunization (d) and number of EAE and control mice per group. **b** Boxplots showing the percentage of CD3+ T cells and CD56dim NK cells expressing EZH2 in the CSF and peripheral blood compartments of multiple sclerosis patients (*n* = 3 for CSF; *n* = 10 for blood). **c** Expression of EZH2 in CD4+ and CD8+ T cells from chronic active lesions of multiple sclerosis patients (*N* = 10). Arrows indicate CD4+ or CD8+ T cells expressing EZH2. Graph shows the percentage of CD4+ and CD8+ T cells expressing EZH2 in CNS lesions. Bars represent mean (standard error of the mean). Statistics: **a** unpaired Student’s *t* test; **b** Mann-Whitney *U* test. *p values < 0.05; **p values < 0.01; ***p values < 0.001. EZH2, enhancer of zeste 2 polycomb repressive complex 2 subunit; Cd3 Cd3e, CD3 antigen, epsilon polypeptide

Based on the gene and protein expression findings, we hypothesized that the decrease of circulating EZH2-positive T cells in untreated multiple patients compared to controls was secondary to the migration of EZH2-positive T cells into the CNS. To evaluate this hypothesis, we first investigated EZH2 expression in the CNS of EAE mice and observed that EZH2 was expressed in spinal cord tissue during EAE, and EZH2 expression levels peaked at the inflammatory phase of the disease (day 16 post-immunization) (Fig. 5a). Interestingly, EZH2 followed a similar temporal pattern of CNS expression to Cd3 in EAE mice, and expression levels for these two genes correlated with each other (Spearman correlation coefficient = 0.63, *p* = 0.002), suggesting a relationship between EZH2 expression and the inflammatory cell infiltrate during EAE (Fig. 5a). We next aimed to extrapolate these findings to patients with multiple sclerosis by determining EZH2 expression in CSF cells from three untreated patients. As shown in Fig. 5b, EZH2-positive T cells were enriched in the CSF compartment, and the percentage of CD3+ T cells expressing EZH2 was significantly increased in the CSF compared to peripheral blood (*p* = 0.007). In contrast, in CD56dim NK cells, a cell subset that also expressed EZH2 (Fig. 4a), the percentage of EZH2-positive cells did not differ between the CSF and blood compartments, indicating a preferential capacity for EZH2-positive T cells to migrate into the CNS. In this line, we finally investigated EZH2 expression in chronic active lesions from 10 multiple sclerosis patients and observed that 8.5% (mean percentage) and 10.9% of the total CD4+ and CD8+ T cells were expressing EZH2 respectively (Fig. 5c). Occasionally, EZH2 expression was also observed in the nuclei of few glial cells, and brain tissues from non-neurological controls were negative for EZH2 expression (data not shown).

### EZH2 blocking downregulates T cell adhesion molecules

**Fig. 6:**
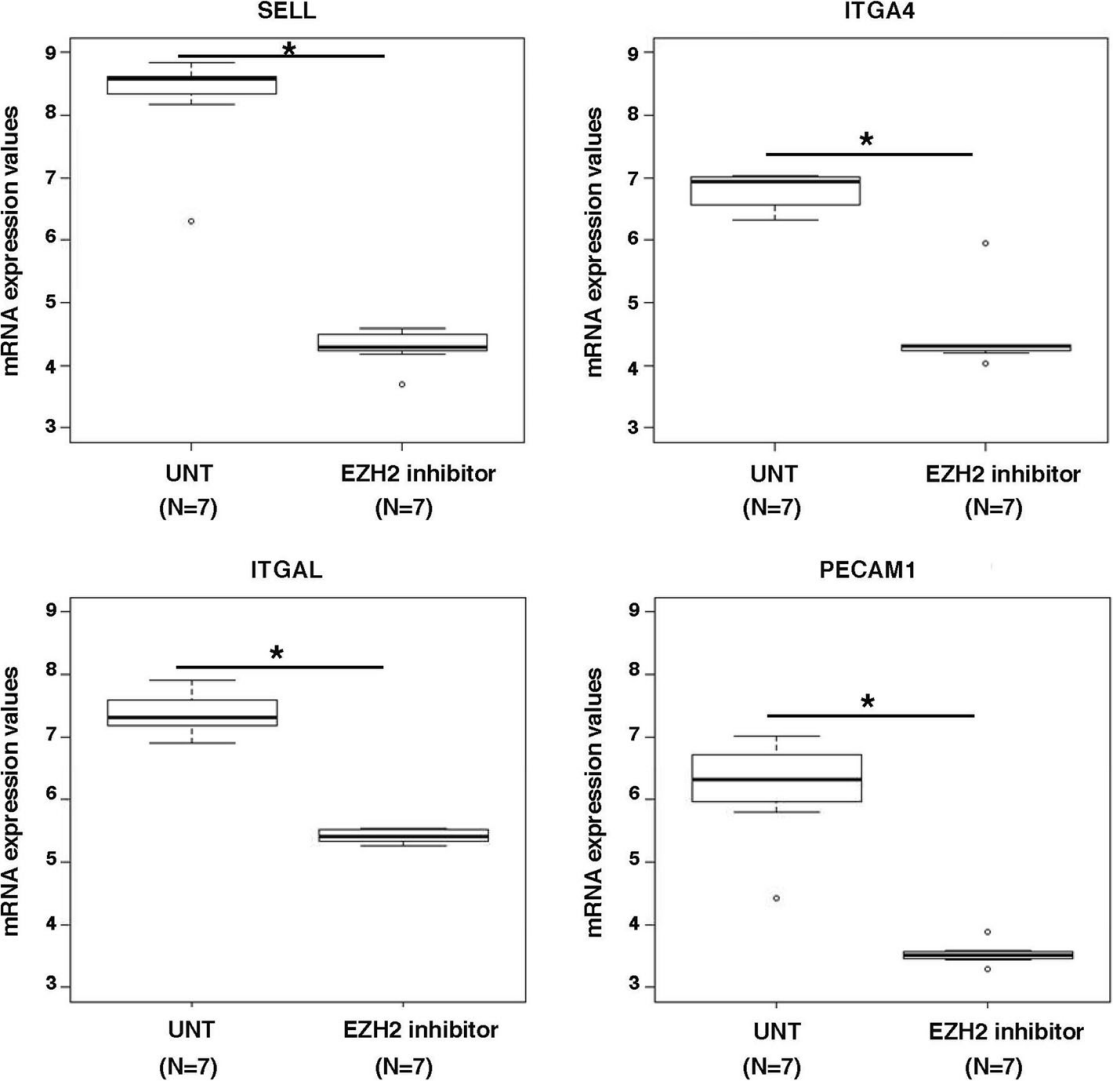
Expression of cell adhesion molecules in PBMC following EZH2 blocking. Boxplots showing expression levels obtained with microarrays for adhesion molecules before and after incubation of PBMC from multiple sclerosis patients with an EZH2 inhibitor for 24 h. The asterisk symbol refers to adjusted *p* values < 0.001. UNT untreated condition, SELL selectin L, ITGA4 integrin subunit alpha 4, ITGAL integrin subunit alpha L, PECAM1 platelet and endothelial cell adhesion molecule 1

As a last step, we incubated in vitro PBMC from seven untreated multiple sclerosis patients with an EZH2 inhibitor in order to investigate the genes modulated by EZH2 and aiming to better understand the role of EZH2 in disease pathophysiology. A total of 6763 genes were significantly up- or downregulated by the effect of the EZH2 inhibitor (with *p* values < 0.05; data not shown). Interestingly, among the top 1% of differentially expressed genes between the untreated and treated conditions, we identified key T cell adhesion molecules that were strikingly downregulated by the EZH2 inhibitor such as selectin L (SELL, also known as CD62L; adjusted *p* value versus the untreated condition = 6.4 × 10^−20^), integrin subunit alpha 4 (ITGA4, also known as CD49D; *p* = 3.6 × 10^−16^), integrin subunit alpha L (ITGAL, also known as CD11A; *p* = 1.1 × 10^−13^), and platelet and endothelial cell adhesion molecule 1 (PECAM1; *p* = 5.2 × 10^−14^) (Fig. 6). These data support a role of EZH2 in the adhesion of circulating T cells.

## Discussion

Extensive literature exists about the role of EZH2 as histone methyltransferase and, particularly, about EZH2 involvement in a wide range of malignant tumors [8, 12, 13] due to its function as epigenetic silencer [14]. The identification of a cytosolic methyltransferase complex containing EZH2 suggested that, in addition to its nuclear role, EZH2 could also be involved in other important cellular processes such as cell adhesion and migration [2]. In an attempt to characterize more in depth the cytosolic role of EZH2, Gunawan et al. [2] recently reported that EZH2 was critical for regulating leukocyte migration to sites of inflammation in EAE mice, findings that opened a potential and attractive link between EZH2 and autoimmune disorders such as multiple sclerosis in which cell adhesion and migration are critical pathogenic mechanisms [15]. Despite this initial publication in the animal model of multiple sclerosis [3], to date, there are no studies of EZH2 in patients with multiple sclerosis. Aiming to explore the role of EZH2 in multiple sclerosis, we first determined mRNA expression levels in PBMC from untreated patients and healthy individuals and observed that EZH2, together with molecules reported to be associated with cytosolic EZH2 such as TLN1 and VAV1 [3], were all downregulated in multiple sclerosis patients regardless of whether they were having relapse-onset or progressive clinical forms, or whether they were in clinical remission or in acute relapse. The decreased expression of EZH2 in PBMC from patients with multiple sclerosis was replicated in an independent validation cohort of patients and controls. These initial observations at the gene expression level suggested an involvement of EZH2 in the disease, which was explored in additional experiments.

We first explored microRNAs, which are known to exert regulatory functions at the posttranscriptional level via binding to the 3′ untranslated region of target mRNAs [16]. In the search for potential microRNAs that on the one hand regulated EZH2 expression and on the other hand were involved in multiple sclerosis, the microRNAs miR-124 and miR-155 emerged as attractive candidates [8, 9]. In this context, expression levels for miR-124 were found increased in demyelinated hippocampi from postmortem brains of multiple sclerosis patients [10]. miR-155 expression was upregulated in peripheral blood monocytes and active lesions from patients [11]. In our study, when expression levels for these two microRNAs were determined in PBMC from a subgroup of untreated multiple sclerosis patients and controls, miR-124 but not miR-155 was significantly upregulated in patients, suggesting a potential functional relationship between decreased EZH2 mRNA expression levels and miR-124 upregulation in patients with multiple sclerosis. These findings warrant future studies to investigate the cell types that contribute to miR-124 upregulation.

In order to explore whether the expression of EZH2 and associated molecules was modulated by commonly used disease-modifying therapies in patients with multiple sclerosis, a cohort of patients treated with interferon-beta, glatiramer acetate, fingolimod, and natalizumab was also included in the study. Treatment with interferon-beta, glatiramer acetate, and natalizumab was associated with increased expression levels of EZH2 and TLN1. By contrast, fingolimod treatment was only associated with upregulated expression of TLN1. Although based on a small number of samples and in spite of the descriptive nature of the experiments, it is tempting to speculate that this finding may be due to the different mechanisms by which these drugs regulate leukocyte migration to the CNS, being the mechanism of action of fingolimod not exerted in peripheral blood but in the lymph nodes where it retains naïve and central memory lymphocytes [17–20]. In view of these data, we hypothesize that the reduction in EZH2 expression observed in untreated patients with subsequent upregulation after treatment may indicate a migration capacity of PBMC expressing EZH2 to the CNS that is inhibited by the effect of treatment. These findings also open a new research avenue to investigate whether the increase in EZH2 expression observed after treatment is associated with the response to therapies and hence may differ between responders and non-responders to each particular therapeutic strategy.

Immunophenotyping of the major PBMC populations revealed restricted EZH2 expression in CD4+ and CD8+ T cells as well as CD56dim NK cells. Similar to the gene expression findings, the percentage of EZH2-positive T cells was reduced in untreated multiple sclerosis patients compared to controls, pointing to a role of EZH2 in this particular cell subset rather than in CD56dim NK cells, which showed similar percentages of EZH2-positive cells in patients and controls. It is worth highlighting that except for one patient and one control, the immunophenotyping cohort included new individuals, and hence, the decrease of EZH2-positive T cells observed in patients can also be considered as a new validation of the EZH2 expression findings at the protein level. Natalizumab, which was selected as control therapy because of its known effects reducing T cell trafficking into the CNS, increased the percentage of EZH2-positive T cells, but, contrary to gene expression results, differences did not reach statistical significance. Interestingly, further T cell immunophenotyping showed heterogeneity in the T cell subsets positive for EZH2 expression. In this context, most CD4+ T cells expressing EZH2 were effector memory T cells, a population that per se has the capacity to migrate to non-lymphoid tissues including the CNS [21]. In contrast, CD8+ T cells positive for EZH2 had a predominant central memory phenotype, suggesting that this particular subset can migrate to the CNS in MS and may later differentiate into effector memory populations in the sites of inflammation. Of note, EZH2-positive cells both in CD4+ and CD8+ T cells may have pathogenic potential through the secretion of TNF-α, a pro-inflammatory cytokine involved in the pathogenesis of multiple sclerosis [22]. Although caution should be taken when considering these data owing to the small sample size and high variability, these findings altogether warrant additional studies to deepen into the pathogenic capacity of T cells expressing EZH2 and explore whether EZH2 may become a therapeutic target in MS patients to reduce disease activity.

The potential for EZH2-positive T cells to migrate to the CNS was first suggested in the EAE study, which showed EZH2 expression in spinal cord tissue from EAE mice following a similar pattern to Cd3 expression over time. Confirmation of the migratory capacity of EZH2-positive T cells was provided by their detection in the CSF and brain lesions from multiple sclerosis patients. Noteworthy, the enrichment for CD4+ and CD8+ T cells expressing EZH2 in the CSF and chronic active lesions compared to the blood compartment suggested that the decrease of EZH2-positive T cells observed in untreated multiple sclerosis patients compared to healthy individuals was secondary to their migration into the CNS. This notion was further supported by the observation that other EZH2-expressing blood cell populations such as CD56dim NK cells, which did not depict differences between patients and controls in blood, were not enriched in the CSF. Although EZH2 expression in brain lesions from multiple sclerosis patients seemed restricted to CD4+ and CD8+ T cells, additional sources of EZH2 expression within the CNS cannot be totally ruled out, as evidenced by EZH2 immunohistochemistry. In contrast, EZH2 expression was not observed in brain tissue from non-neurological controls, a finding that confers specificity for EZH2 expression in the CNS of multiple sclerosis patients.

## Conclusions

Finally, the implication of EZH2 in T cell adhesion, an important step for cell migration, was supported by the microarray findings conducted after EZH2 blocking with a histone deacetylase inhibitor that regulates EZH2 expression [23], which showed striking downregulation of cell adhesion molecules expressed in T cells such as SELL [24], ITGA4 [25], ITGAL [26], and PECAM1 [27]. The aggregate results from the study suggest a role for EZH2 in the migration of T cells into the CNS in patients with multiple sclerosis and also suggest a potential pathogenic capacity of EZH2-positive T cells that will need to be explored more in depth in future studies.

## Additional file

Additional file 1: **Table S1.** Demographic and clinical characteristics of the multiple sclerosis patients and HC included for the determination of microRNA expression levels. (DOC 36 kb)

## Abbreviations

EAE: Experimental autoimmune encephalomyelitis
EZH2: Enhancer of zeste 2 polycomb repressive complex 2 subunit
GM-CSF: Granulocyte/macrophage colony-stimulating factor
H3K27: Histone H3 at lysine 27
HC: Healthy controls
ITGA4: Integrin subunit alpha 4
ITGAL: Integrin subunit alpha L
KB: Klüver-Barrera
mRNA: Messenger RNA
MS: Multiple sclerosis
PBMC: Peripheral blood mononuclear cells
PECAM1: Platelet and endothelial cell adhesion molecule 1
PMA: Phorbol 12-myristate 13-acetate
PPMS: Patients with primary progressive multiple sclerosis
RRMS: Relapsing-remitting multiple sclerosis
SAHA: Suberoylanilide hydroxamic acid
SELL: Selectin L
TLN1: Talin 1
TNF: Tumor necrosis factor
