# Supplementary material for: Circulating EZH2-positive T cells are decreased in multiple sclerosis patients
Source: J Neuroinflammation. 2018 Oct 26;15:296. doi: 10.1186/s12974-018-1336-9 (PMC6202809; doi:10.1186/s12974-018-1336-9)
Supplement: Supplementary file 1 — Table S1. Demographic and clinical characteristics of the multiple sclerosis patients and HC included for the determination of microRNA expression levels. (DOC 36 kb) [file 12974_2018_1336_MOESM1_ESM.doc]

**Supplementary Content**

**Supplementary Table 1.** Demographic and clinical characteristics of the multiple sclerosis patients and HC included for the determination of microRNA expression levels.

| Baseline characteristics | HC | MS patients |
| --- | --- | --- |
| N | 18 | 21 |
| Age (years) | 29.6 (6.9) | 35.3 (10.2) |
| Female/male (% women) | 13/5 (72.2) | 11/10 (52.4) |
| Duration of disease (years) | - | 6.1 (6.9) |
| Clinical form (number of patients) | - | 15 RRMS/6 SPMS |
| EDSS^a^ | - | 2.8 (1.0-3.8) |
| Numbers of relapses^b^ | - | 1.8 (1.1) |

Data are expressed as mean (standard deviation) unless otherwise stated. ^a^Data are expressed as mean (interquartile range). ^b^Refers to the number of relapses in the two years before blood collection. HC: healthy controls. RRMS: relapsing-remitting multiple sclerosis. SPMS: secondary progressive multiple sclerosis.
